# Species Pervasiveness Within the Group of Coagulase-Negative Staphylococci Associated With Meat Fermentation Is Modulated by pH

**DOI:** 10.3389/fmicb.2018.02232

**Published:** 2018-09-19

**Authors:** Despoina Angeliki Stavropoulou, Hannelore De Maere, Alberto Berardo, Bente Janssens, Panagiota Filippou, Luc De Vuyst, Stefaan De Smet, Frédéric Leroy

**Affiliations:** ^1^Research Group of Industrial Microbiology and Food Biotechnology, Faculty of Sciences and Bioengineering Sciences, Vrije Universiteit Brussel, Brussels, Belgium; ^2^Research Group for Technology and Quality of Animal Products, KU Leuven, Technology Campus Ghent, Ghent, Belgium; ^3^Laboratory for Animal Nutrition and Animal Product Quality, Department of Animal Production, Ghent University, Ghent, Belgium

**Keywords:** *Staphylococcus xylosus*, *Staphylococcus equorum*, *Staphylococcus saprophyticus*, starter cultures, dry fermented sausages, pH

## Abstract

During spontaneous meat fermentations, *Staphylococcus equorum*, *Staphylococcus saprophyticus*, and *Staphylococcus xylosus* are generally the most prevailing species within the communities of coagulase-negative staphylococci (CNS). There is an interest to introduce CNS isolates from artisan-style spontaneous meat fermentations as starter cultures in more industrialized processes, as to confer additional quality benefits. However, staphylococcal competitiveness within the meat matrix is affected by the processing conditions, which vary considerably among product types. A major factor of variability relates to the intensity of acidification, driven by the concentration of added carbohydrates. The effect of pH on CNS prevalence was studied in both a mince-based meat fermentation model and in fermented sausages produced on pilot scale. Roughly, from all experiments combined, it appeared that a pH of 5.3 corresponded with a breakpoint for CNS selection. Above this value, a general prevalence by *S. xylosus* was found, even overruling the addition of starter cultures consisting of *S. equorum* and *S. saprophyticus* strains. At pH values below 5.3, *S. xylosus* was also accompanied by *S. equorum* (following a mild pH drop) and *S. saprophyticus* (following a stronger pH drop). Still, addition of starter cultures affected the volatile profile compared to the control batch, even if those starter cultures were not able to dominate during the ripening process. This study nonetheless provides a warning for an overly confident use of specific CNS species as starter cultures, especially when in a given processing context the prevailing conditions do not allow superior growth compared to the CNS from the background microbiota.

## Introduction

Coagulase-negative staphylococci (CNS) are fundamental to the process of meat fermentation, whereby their metabolic activities contribute to the development of qualitative end-products (Sánchez Mainar et al., 2017). In contemporary meat processing plants, the production of fermented meats is now habitually performed by addition of a starter culture based on a mixture of selected lactic acid bacteria (LAB) and CNS strains to allow more control over the process (Leroy et al., 2006). For some products, the artisan-type technique of spontaneous fermentation is still in use, albeit rarely on an industrial scale (Talon et al., 2007; Coton et al., 2010; Janssens et al., 2013; Leroy et al., 2015). In spontaneously fermented variants, *Staphylococcus equorum*, *Staphylococcus saprophyticus*, and/or *Staphylococcus xylosus* are usually the (co)dominant CNS species, besides a wide diversity of other minor species (Mauriello et al., 2004; Corbière Morot-Bizot et al., 2006; Iacumin et al., 2006, 2012; Drosinos et al., 2007; Marty et al., 2012; Greppi et al., 2015; Pisacane et al., 2015; Ratsimba et al., 2017). The species composition of staphylococcal consortia in fermented meats is nevertheless difficult to predict, as this largely depends on the processing parameters applied (Leroy et al., 2014). The acidification level is one of the main factors shaping the CNS microbiota during meat fermentation, whether they are added as starter cultures or not (Stavropoulou et al., 2018a,b). For instance, *S. xylosus* often dominates in Southern-European type of fermented sausages that are characterized by mild pH values (Cocolin et al., 2001a; Rossi et al., 2001; Iacumin et al., 2006; Di Cagno et al., 2008; Greppi et al., 2015), whereas *Staphylococcus carnosus* is uncommon in spontaneous fermentations and seems to be more adapted to highly acidified sausages (Stahnke et al., 2002; Stavropoulou et al., 2018a). The speed and extent of acidification are set by the degree of carbohydrates available, the fermentation temperature, and the use of acidifying LAB strains. Surface molding, common in many types of fermented sausages, also affects the pH through the consumption of lactic acid and, thus, modulates staphylococcal composition as well (Janssens et al., 2013).

With respect to starter culture formulation, *S. carnosus* and/or *S. xylosus* are the two CNS species that are widely used by the meat fermentation industry and which have been studied thoroughly with respect to their technological properties (Beck et al., 2002, 2004; Olesen et al., 2004; Tjener et al., 2004; Casaburi et al., 2005; Leroy et al., 2006, 2017; Rosenstein et al., 2009) and their evolution during meat fermentation (Di Maria et al., 2002; Corbière Morot-Bizot et al., 2007; Stavropoulou et al., 2018a). Although other CNS species than the latter two have been studied for their technologically relevant features (Villani et al., 2007; Talon et al., 2008; Bonomo et al., 2011; Casquete et al., 2011; Fonseca et al., 2013a,c; Prpich et al., 2016), usually only a few studies monitor their prevalence after inoculation as novel starter culture candidates (Corbière Morot-Bizot et al., 2007; Talon et al., 2008; Fonseca et al., 2013c; Sánchez Mainar and Leroy, 2015). The ability of starter cultures to persist during meat fermentation with respect to the background microbiota depends on the processing parameters and this is not always guaranteed, for instance because of the different degrees of acidification driven by the carbohydrate concentrations added (Villani et al., 2007; Sánchez Mainar and Leroy, 2015; Stavropoulou et al., 2018a).

In the current study, the effect of the acidification level on CNS communities during meat fermentation was investigated in a mince-based meat model system as well as during actual dry fermented sausage production on pilot scale. Focus was placed on the relative pervasiveness of *S. equorum*, *S. saprophyticus*, and *S. xylosus*, both with respect to their spontaneous outgrowth and when added as starter cultures.

## Materials and Methods

### Bacterial Strains, Media, and Inoculum Build-Up

The three CNS strains, namely *S. equorum* DFL-S19, *S. saprophyticus* FPS1, and *S. xylosus* 2S7-2, and the LAB strain (*Lactobacillus sakei* CTC 494) were used in this study, originating from the culture collection of the research group of Industrial Microbiology and Food Biotechnology (Vrije Universiteit Brussel, Brussels, Belgium). The CNS strains were selected based on their ability to produce volatile compounds during an *in vitro* assay (Stavropoulou et al., 2015). They were stored at -80°C in glycerol-containing (25%, v/v) brain heart infusion (BHI) medium (Oxoid, Basingstoke, Hampshire, United Kingdom) for the CNS or de Man-Rogosa-Sharpe (MRS) medium (Oxoid) for the LAB. For the inoculum build-up, all strains were propagated twice in the appropriate media and incubated at 30°C for 12 h. The precultures were then inoculated (1%, v/v) into BHI or MRS medium for the final inoculum at 30°C for 12 h. Cell pellets were collected by centrifugation (8041 × *g* at 4°C for 20 min) and used as inoculum for the sausage preparation after re-suspension in saline solution (0.85%, m/v, NaCl; VWR International, Darmstadt, Germany).

### Meat Fermentations by Means of a Mince-Based Meat Model

The effect of the acidification level on the CNS community dynamics was studied in a mince-based meat model system that was used to carry out the meat fermentations. The total prepared meat batter consisted of fresh pork mince (3 kg), supplemented with 2.5% of sodium chloride (m/m; VWR International), 500 ppm of ascorbic acid (Sigma-Aldrich, Steinheim, Germany), 200 ppm of sodium nitrate (VWR International), and 200 ppm of MnSO_4_⋅4H_2_O to stimulate LAB growth (VWR International). The meat batter was inoculated with *L. sakei* CTC 494 at a level of approximately 6.0 log of colony forming units (cfu) per gram. Two different batches were prepared by adding different glucose (VWR International) concentrations to achieve a strong (0.7%, m/m) or mild (0.1%, m/m) pH decrease during the fermentation courses. In addition, for each glucose level a different temperature profile was applied to obtain two distinct pH profiles of strong and mild acidification, corresponding with what is usually applied in North and South Europe, respectively (**Table 1**). The meat mixture of each batch was stuffed into 60-ml plastic containers (approximately 100 g per container; VWR International) to enable fermentation in the absence of air. The containers were placed in a water bath coupled to a cryostat (Frigomix 3000T; Sartorius, Goettingen, Germany) and the fermentations were followed for 7 days. Bacterial counts and pH measurements were performed at days 1, 2, 3, 4, and 7. For each time point, three randomly selected containers were selected for analysis.

**Table 1:** Temperature profiles used for the meat fermentation experiments by means of a mince-based meat model.

Profile/Time (days)	Strong acidification (0.7% glucose, m/m)	Mild acidification (0.1% glucose, m/m)
	Temperature (°C)	
0	25	20
1	25	20
2	25	20
3	20	18
4	18	16
5	16	14
6	14	14
7	14	14

### Pilot-Scale Production of Dry Fermented Sausages

Fermented sausages were produced in a pilot-scale plant at the Technology Campus Ghent (KU Leuven, Ghent, Belgium). For their preparation, the following basic ingredients were mixed (in %, m/m): cutter lean pork (70.5), pork backfat (27.0), sodium chloride (2.5), sodium ascorbate (0.05), and sodium nitrate (0.015). The meat batter was stuffed into collagen casings of 50 mm diameter (Naturin, Weinheim, Germany), which were dipped in a sorbate solution. The total sausage batter was around 30 kg, whereas the initial mass of each fermented sausage was approximately 200 g. The sausages were ripened in a climate chamber (Kerres Anlagensysteme, Backnang, Germany) for 28 days. The fermentation step lasted 2 or 4 days for the experiments without or with carbohydrates added, respectively (see below), i.e., before the pH started to level off. The fermentation step was performed at 24°C and a relative humidity of 94%. For the ripening process, the temperature was decreased to 12°C and the relative humidity was adjusted to 80% for the last 10 days.

Two different experiments were performed on pilot-scale level. In a first experiment, the aim was to validate the results obtained from the mince-based meat model system in a more realistic set-up. In this case, the meat batter was inoculated with *L. sakei* CTC 494 solely (6.0 log cfu/g), using two different carbohydrate concentrations added initially (0.25 and 0.70% of glucose, m/m), to be able to test the impact of the acidification level on the dynamics of the autochthonous CNS communities in two batches differing in pH evolution.

In a second experimental set-up, the fate of the three different CNS strains applied (*S. equorum* DFL-S19, *S. saprophyticus* FPS1, and *S. xylosus* 2S7-2) was investigated during a specific sausage fermentation process characterized by a low acidification degree (i.e., no added carbohydrates), as to meet the conditions under which the corresponding species were expected to be most robust. Four different batches were prepared, of which three were based on the inoculation of one of the three CNS strains under investigation and a fourth one consisted of a control batch without the CNS strains. *L. sakei* CTC 494 was added to all four batches. All strains were inoculated at a level of approximately 6.0 log cfu/g.

Three sausages were analyzed per batch and per time point for each experimental set-up. For the first experiment, each variant was produced three times. Samples were taken for the bacterial enumeration and the pH measurements after inoculation (day 0), at the end of the fermentation step (day 2 or 4), and at the end of the ripening stage (28 days). The CNS communities were analyzed at the end of the ripening stage. For the second experiment, additional samples were taken after 1, 7, 14, and 21 days and survival of the starter culture was followed throughout the production process (2, 14, and 28 days). The experiment was repeated two times.

### pH Measurements

The pH of each sample of the mince-based meat model and pilot-scale meat fermentation experiments was measured using a DY-P10 pH meter (Sartorius). Regarding the mince-based meat model fermentation samples, one randomly selected container was used, and triplicate measurements were performed. For the pilot-scale sausage production samples, the pH measurements were performed for three randomly selected sausages per experimental variant.

### Enumeration and Isolation of Microorganisms

Either 12 g of the mince-based meat model fermentation samples or 25 g of the pilot-scale sausage production samples were aseptically transferred into a stomacher bag (Seward, Worthing, West Sussex, United Kingdom) together with 108 or 225 ml of maximum recovery diluents [sterile solution of 0.85% (m/v) NaCl (VWR International) and 0.1% (m/v) bacteriological peptone (Oxoid)], respectively. This mixture was homogenized at high speed for 2 min in a stomacher (Stomacher 400; Seward). Appropriate decimal dilutions in saline were prepared and spread on MRS agar and mannitol salt-phenol-red agar (MSA; VWR International) media. The agar media were incubated at 30°C for 48–72 h and the counts were determined from agar media containing 30–300 colonies. Next, 10–30% of the colonies were randomly picked up to analyze the LAB and CNS communities. The MSA- and MRS agar-derived colonies were transferred into BHI and MRS broth media, respectively, and incubated at 30°C overnight. The cultures obtained were stored at -80°C in cryovials, containing the appropriate media and 25% (v/v) of glycerol, and used for DNA extraction.

### Classification and Identification of Bacterial Isolates Through (GTG)_5_-PCR Fingerprinting of Genomic DNA

Genomic DNA extraction from cell pellets obtained by microcentrifugation at 13,000 rpm of 1.5 ml of an overnight LAB and CNS culture was performed with a Nucleospin 96 tissue kit (Macherey-Nagel, Düren, Germany), according to the manufacturer’s instructions. Prior to extraction, the cell pellets were washed with Tris-ethylene diaminotetraacetic acid (EDTA)-sucrose buffer [TES buffer; 50 mM Tris base (Calbiochem, Darmstadt, Germany), 1 mM EDTA (Sigma-Aldrich), and 6.7% (m/v) sucrose (VWR International), pH 8.0]. Subsequently, (GTG)_5_-PCR fingerprints of the genomic DNA were obtained, followed by image analysis, as described previously (Braem et al., 2011). Numerical analysis of the fingerprints obtained was performed with BioNumerics 5.1 software (Applied Maths, Sint-Martens-Latem, Belgium). Confirmation of the species identity assigned to each LAB and CNS cluster was done by sequencing of the 16S rRNA and *rpoB* genes of representative isolates, respectively, as described previously (Braem et al., 2011).

### Determination of Volatile Compounds

Volatile compounds produced during the second pilot-scale experiment were measured by solid-phase microextraction (SPME) and gas chromatography coupled to mass spectrometry (GC-MS). Samples were prepared in triplicates in headspace vials containing 5 g of meat sample and 1 g of sodium chloride to enhance volatility (Kolb and Ettre, 2006). Analyses were performed with an Agilent 6890 gas chromatograph (Agilent Technologies; Santa Clara, CA, United States), with a MPS2 Gerstel autosampler (Gerstel GmbH & Co., KG, Mülheim-an-der-Ruhr, Germany), and coupled to an Agilent 5973N mass spectrometer (Agilent Technologies). The capillary column used was a J&W 122-7333 DB-WAXetr (30 m × 0.25 mm × 0.50 μm; Agilent Technologies) and the SPME device (Supelco, Bellefonte, PA, United States) was equipped with a 75-μm divinylbenzene/carboxen/polydimethylsiloxane (DVS/CAR/ PDMS) fiber. Helium was used as carrier gas with a flow rate of 1.0 ml/min. Samples were equilibrated by agitation at 60°C for 40 min prior to injection. The injection port was set in splitless mode (bake-out of the needle at 260°C for 10 min, incubation at 60°C for 5 min, and extraction for 30 min). The injection volume was set at 1 ml, at a rate of 500 μl/s. The oven temperature program consisted of an initial step at 28°C for 3 min, followed by a linear increase from 28 to 220°C at 20°C/min. Finally, the temperature remained constant at 220°C for 5 min. The temperature of the transfer line was held at 280°C. Detection was done by an MS detector (ionization energy 70 eV, 4.1 scans/s, source 230°C, scan range 29–250 m/z). Identification of the peaks was done by comparison with standard compounds that were injected separately and with library data (NIST 08^1^). The peak areas of the examined compounds were considered only in case they exceeded three times the baseline signal (Aguinaga et al., 2008). All values were expressed as peak areas.

### Statistics

Using SPSS version 20 (IBM Corporation, Armonk, NY, United States), a Kruskal–Wallis test was used to check for overall significant differences in metabolite production between batches, followed by Mann–Whitney tests as non-parametric two-sample tests for the comparison of values.

## Results

### Meat Fermentations by Means of a Mince-Based Meat Model

**FIGURE 1:**
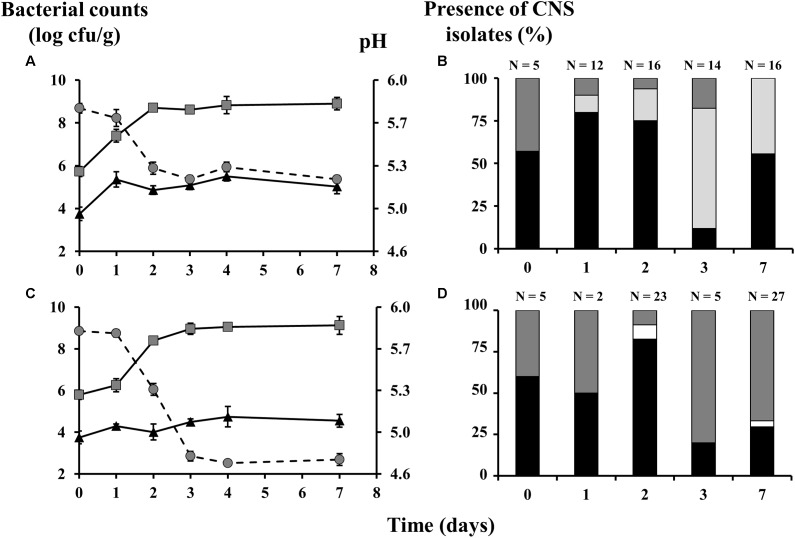
Counts of lactic acid bacteria (gray squares) and coagulase-negative staphylococci (CNS; black triangles), as well as the pH profiles (gray circles) and the species diversity of the staphylococcal communities of meat fermentations by means of mince-based meat models, inoculated with *Lactobacillus sakei* CTC 494, and with mild **(A,B)** or strong **(C,D)** acidification profiles. The presence of CNS isolates is displayed as relative abundance, calculated based on number of isolates (N). The bacterial species were identified as *Staphylococcus xylosus* (black bars), *Staphylococcus saprophyticus* (dark gray bars), *Staphylococcus equorum* (light gray bars), and *Staphylococcus carnosus* (white bars).

Due to the two different initial glucose concentrations added in combination with the applied temperature profiles, two different acidification profiles were obtained (**Figure 1**). For the meat batter with the mild acidification profile, the pH dropped from 5.8 to around 5.2 after 3 days of fermentation, after which it more or less stabilized (**Figure 1A**). This evolution paralleled the bacterial counts measured on MRS agar, which increased from 5.73 to 8.71 log cfu/g after 3 days of fermentation and then remained stable till the end of the experiment. The MSA-derived bacterial counts increased from 3.75 to 5.36 log cfu/g after 1 day of fermentation and subsequently fluctuated between the values of 4.87 and 5.51 log cfu/g. During the fermentation with the strong acidification set-up, the pH decreased to 4.7 after 4 days of fermentation and remained stable till the end of the experiment (**Figure 1C**). After fermentation, the LAB population had increased to 9.05 log cfu/g and then remained around this value, which was slightly higher than in the mildly acidified variants. The MSA counts slowly increased to their maximum of 4.74 log cfu/g after 4 days of fermentation and were estimated at 4.55 log cfu/g after 7 days.

The initial CNS population of the meat was composed of *S. xylosus* (57% of the isolates; 99% sequence identity, accession number LN554884.1) and *S. saprophyticus* (43% of the isolates; 99% sequence identity, accession number AP008934.1). In the mildly fermented variants, *S. xylosus* became the most prevalent CNS species during the first 2 days of fermentation, during which *S. saprophyticus* and *S. equorum* (98% sequence identity, accession number LM651926.1) were subdominantly present (**Figure 1B**). After 3 days of fermentation, however, *S. equorum* took over and represented 86% of the CNS community. After 7 days, *S. equorum* and *S. xylosus* reached more or less equal representation levels of 44 and 56%, respectively. In the more acidified variants, *S. xylosus* and *S. saprophyticus* were the two major CNS species and *S. equorum* was no longer found (**Figure 1D**). Instead, *S. carnosus* (98% sequence identity, accession number AM295250.1) emerged but was only present in low levels after two (9%) and 7 days (4%) of fermentation. Whereas *S. xylosus* and *S. saprophyticus* were more or less equally present at the beginning of the fermentation, it was ultimately *S. saprophyticus* that became the leading species (67% of the staphylococcal isolates) after 7 days.

### Pilot-Scale Dry Fermented Sausage Production

#### Effect of Acidification Profile

During the first experimental pilot-scale sausage production set-up, two different initial glucose levels were used to achieve two distinct acidification profiles generated by spontaneous fermentation (**Table 2**). When 0.25% (m/m) of glucose was added, the pH dropped from 5.69 to 5.23 after 4 days of fermentation and then increased at the end of ripening to 5.92. For the meat batters with 0.70% (m/m) of added glucose, the pH decreased from 5.69 to 4.89 after 4 days of fermentation and then slightly increased after 28 days to 4.97. For all batches, the MRS agar counts were 6.45 log cfu/g after inoculation and the ones on MSA equaled to 3.46 log cfu/g. The LAB population followed a similar pattern in both batches independent of the level of acidification, increasing at the end of fermentation to 7.87 log cfu/g and further at 8.11 log cfu/g by the end of the ripening stage. On the other hand, the pH profile had an effect on the bacterial counts enumerated on MSA. For the batches with a low glucose concentration, the MSA counts increased throughout the production process, reaching 5.64 log cfu/g after 4 days of fermentation and 5.94 log cfu/g after 28 days. In the more acidified variants, the bacterial counts on MSA increased up to 4.66 log cfu/g at the end of the fermentation step and afterward reached their maximum value of 4.89 log cfu/g by the end of the ripening stage. However, based on the bacterial isolates retrieved from MSA, the corresponding communities consisted mostly of non-further identified isolates that were not belonging to the CNS group as they yielded atypical colony morphology and could not be fingerprinted by (GTG)_5_-PCR. *S. xylosus* (9% of the MSA isolates) and *S. saprophyticus* (3%) were found in the sausages with a mild pH decrease; for the sausages with the pH values falling below 5.00 after 28 days, no staphylococcal isolates were retrieved.

**Table 2:** Evolution of the pH and the bacterial counts (log cfu/g; mean and standard deviations) on MRS agar and MSA associated with fermented sausages produced in triplicate on pilot-scale, in the presence of two different initial glucose concentrations resulting in two distinct acidification profiles.

		Mild acidification		Strong acidification	
Time (days)	0	4	28	4	28
pH	5.69 ± 0.07	5.23 ± 0.02	5.92 ± 0.04	4.89 ± 0.01	4.97 ± 0.05
MRS agar	6.45 ± 0.08	7.87 ± 0.18	8.11 ± 0.18	7.93 ± 0.21	8.05 ± 0.26
MSA	3.46 ± 0.31	5.64 ± 0.05	5.94 ± 0.06	4.66 ± 0.05	4.89 ± 0.06

#### Comparison of Different CNS Starter Cultures

**FIGURE 2:**
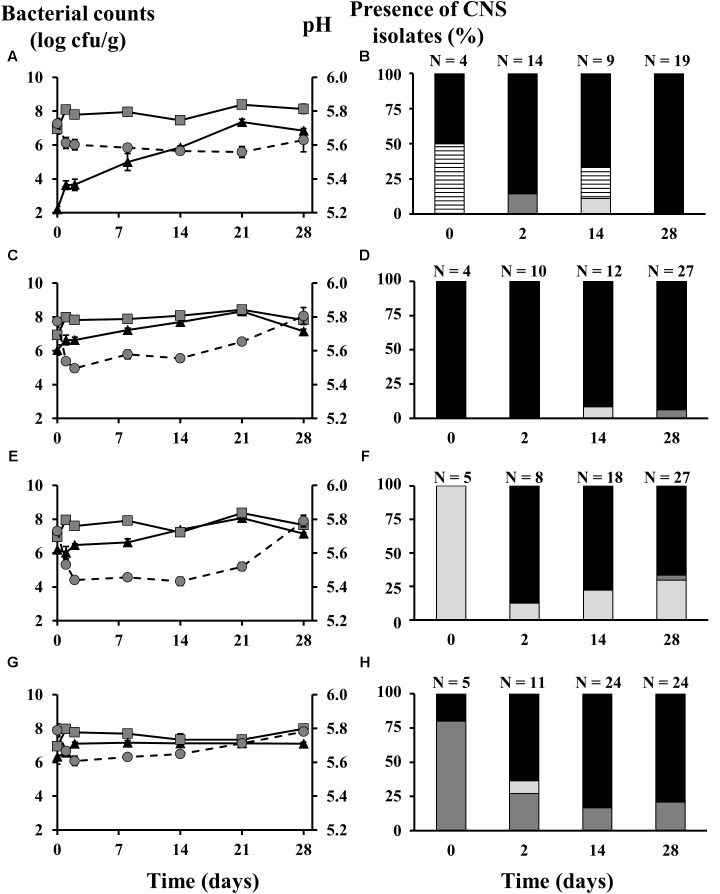
Counts of lactic acid bacteria (gray squares) and coagulase-negative staphylococci (CNS; black triangles), as well as the pH profiles (gray circles) and the species diversity of the staphylococcal communities of fermented sausages produced on pilot-scale inoculated with different CNS strains: **(A,B)** *Lactobacillus sakei* CTC 494 without CNS strains added; **(C,D)** *Staphylococcus xylosus* 2S7-2 and *L. sakei* CTC 494; **(E,F)** *Staphylococcus equorum* DFL-S19 and *L. sakei* CTC 494; **(G,H)** *Staphylococcus saprophyticus* FPS1 and *L. sakei* CTC 494. The presence of CNS isolates is displayed as relative abundance, calculated based on number of isolates (N). The bacterial species were identified as *Bacillus* spp. (white bars with lines), *S. xylosus* (black bars), *S. equorum* (light gray bars), and *S. saprophyticus* (dark gray bars).

In the second pilot-scale sausage production experiment, no carbohydrates were added, as to meet artisan-type conditions under which CNS are normally within a comfortable pH range. The initial LAB counts, as estimated on MRS agar at a level 6.80 log cfu/g, similarly reached a ceiling level of around 8.0 log cfu/g for all batches (**Figure 2**). As a result of this poor LAB growth, the pH of all batches remained above 5.40. The pH evolution for the different batches followed roughly the same pattern, decreasing during the fermentation course of 2 days and then increasing till the end of the ripening stage, especially for the batches inoculated with CNS starter cultures. The latter batches reached a final pH of about 5.80, whereas the batches that were not inoculated with CNS only reached 5.56. Regarding the presumable CNS counts (MSA), the control batches without added CNS had an initial population of 2.20 log cfu/g, which gradually increased to 7.36 log cfu/g after 21 days and subsequently decreased to 6.85 log cfu/g at the end the sausage production process. The initial population of all the inoculated batches was around 6.20 log cfu/g. For the fermented sausages inoculated with *S. equorum* and *S. xylosus*, the MSA counts were continuously increasing till day 21 when they reached their maxima of 8.08 log cfu/g and 8.33 log cfu/g, respectively, and then both decreased to 7.15 log cfu/g. The MSA counts of the batches inoculated with *S. saprophyticus* remained more or less stable from day 2 till the end of the ripening stage (7.10 log cfu/g).

When exploring the CNS species diversity represented in the pool of the MSA isolates obtained, differences were seen between the four inoculation strategies (**Figure 2**). During the fermentations initiated with only a LAB starter culture and no added CNS (**Figure 2B**), *S. xylosus* strain(s) emerged from the background microbiota and prevailed throughout the sausage production process. The initial MSA-associated population of the meat was shared between *S. xylosus*, as the only retrieved CNS species, and *Bacillus* spp. (96% sequence identity, accession number KX011931.1). The latter was also isolated after 14 days and disappeared after that. Occasional presence of *S. equorum* and *S. saprophyticus* at levels of 11% (day 14) and 14% (day 2), was also found. However, at the end of the ripening stage, *S. xylosus* was the only CNS species identified. Similar results were obtained from the batch inoculated with *S. xylosus* 2S7-2 (**Figure 2D**). Based on the (GTG)_5_-PCR fingerprinting, it was unclear whether the isolates originated from the background microbiota or the starter culture added. In addition, *S. equorum* and *S. saprophyticus* were again present in minor proportions (below 8%) at days 14 and 28, respectively. When *S. saprophyticus* FPS1 (**Figure 2F**) or *S. equorum* DFL-S19 (**Figure 2H**) were added as starter culture strains, both species managed to survive during the fermentation until day 28 but were not very competitive, since *S. xylosus* was once more the prevailing species in both cases.

#### Volatile Compounds

**FIGURE 3:**
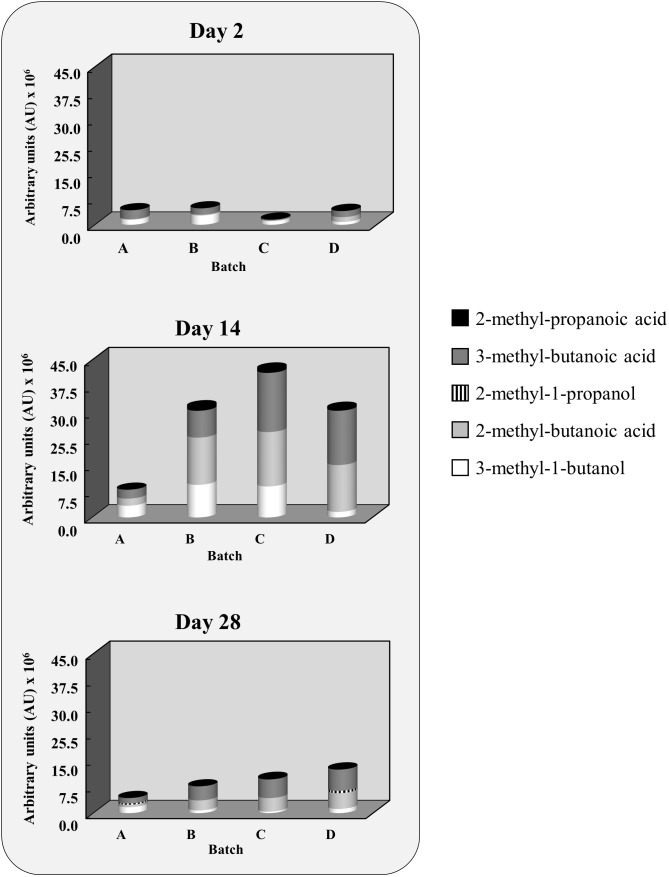
Evolution of the area of the volatile compounds resulting from branched-chain amino acid conversion after 2, 14, and 28 days. Batches with (A) no CNS added or inoculated with (B) *Staphylococcus xylosus* 2S7-2, (C) *Staphylococcus equorum* DFL-S19, or (D) *Staphylococcus saprophyticus* FPS1.

The main volatiles produced during the second pilot-scale experiment originated from physicochemical reactions, relating mostly to lipid autoxidation, as well as from the bacterial conversion of carbohydrates, amino acids, and fatty acids (**Supplementary Table S1**). The evolution of the volatile compounds arising from branched-chain amino acid metabolism, namely 3-methyl-butanoic acid, 2-methyl-butanoic acid, 2-methyl propanoic acid, 2-methyl-propanol, and 3-methyl-butanol, is depicted in **Figure 3**. Differences were found between the batches after 14 days for 3-methyl-butanol (*p* = 0.04), 3-methyl-butanoic-acid (*p* = 0.04), 2-methyl-butanoic acid (*p* = 0.04), and 2-methyl-propanoic acid (*p* = 0.02). At the end of the ripening (28 days), inter-batch differences were limited to 2-methyl-butanoic acid (*p* = 0.02) and 2-methyl-propanoic acid (*p* = 0.04).

After 14 days, the level of 3-methyl-butanol in the batch inoculated with *S. xylosus* 2S7-2 did not differ significantly from the one with *S. equorum* DFL-S19 but was higher than in the control (*p* = 0.05) and the batch inoculated with *S. saprophyticus* FPS1 (*p* = 0.05). The level of its corresponding carboxylic acid, 3-methyl-butanoic acid, did not differ between the various inoculated batches but was always higher than in the control after 14 days (*p* = 0.05). Isoleucine-derived 2-methyl-butanoic acid was also higher in all inoculated batches than in the control after both 14 days (*p* = 0.05) and 28 days (*p* = 0.05). Finally, valine-derived 2-methyl-propanoic acid was higher in all inoculated batches than in the control after 14 days (*p* = 0.05), but not after 28 days. At this stage, only *S. equorum* DFL-S19 displayed still higher levels, not only with respect to the control (*p* = 0.05) but also compared to the other inoculated batches (*p* = 0.05).

## Discussion

*Staphylococcus equorum*, *S. saprophyticus*, and *S. xylosus* are usually the most prevalent CNS species in spontaneously fermented sausages of the Mediterranean type (García-Varona et al., 2000; Papamanoli et al., 2002; Drosinos et al., 2005; Fonseca et al., 2013b; Greppi et al., 2015; Pisacane et al., 2015). The present study contributes to a further validation of this typical association, related to fermented sausages that have low to mild acidification profiles. The experiment using fermented mince-based meat models without added CNS indicated that *S. xylosus* became the leading CNS species during the first days of fermentation and was then accompanied by either *S. equorum* in the mildly acidified batches and by *S. saprophyticus* in the most acid variants. It has indeed been suggested previously that the latter CNS species is somewhat more fit at low pH values than *S. equorum* (Janssens et al., 2013). Note also that a minor fraction of *S. carnosus* emerged in the batches with the lowest pH, as this CNS species is known for its robustness toward acidic conditions but poor adaptation to higher pH values (Stavropoulou et al., 2018a). Comparison of the evolution of the bacterial species diversity with the pH dynamics further suggested that *S. xylosus* was the most competitive CNS species within the initial pH window of fermentation (pH 5.3–5.8), but that a pH of 5.3 served as a breakpoint after which either *S. equorum* or *S. saprophyticus* took over, depending on the extent of the further pH drop. Of course, it needs to be taken into account that the decrease of temperature from 20 to 18°C also may have had a modulating effect, taking into account that *S. equorum* is rather well adapted to low temperatures, provided that the pH is not too low (Mauriello et al., 2004; Leroy et al., 2009).

When investigating the effect of pH in fermented sausages on pilot-scale production level, a pH drop below 5.3 resulted overall in a poor growth of the CNS communities. At the end of the ripening stage, the duo of *S. xylosus* and *S. saprophyticus* was still found in the moderately acidified batches, albeit in minor proportions, but no CNS were retrieved from the batches in which the pH dropped below 5.0 during fermentation. The degree of acidification does indeed not only shape the composition of the staphylococcal communities during meat fermentations but also their levels (Janssens et al., 2013; Stavropoulou et al., 2018a,b). As a result, the CNS communities in Southern-European fermented meat products (Iacumin et al., 2012; Fonseca et al., 2013b; Aquilanti et al., 2016) provide a potentially important contrast to the ones in highly acidified Northern-European variants in which CNS are limited in terms of both numbers and diversity and thus potentially also with respect to quality-yielding functionalities (Ravyts et al., 2010).

When the acidity stress on the CNS communities was minimized by preparing fermented sausages without added carbohydrates, to limit the pH drop by the LAB, the fate of the three CNS species upon addition as starter culture strains revealed two clear patterns. First, superiority of *S. xylosus* within this very mild pH context (pH > 5.4 at all times) was seen in all batches, either due to the background microbiota (in the absence of the starter culture strain *S. xylosus* 2S7-2) or to the added starter culture strain *S. xylosus* 2S7-2. In the latter case, the applied methodology did not allow to differentiate the added strain of *S. xylosus* from *S. xylosus* strains within the background microbiota. A limitation of the (GTG)_5_-PCR classification and identification technique consists of its suboptimal resolution to differentiate on intraspecies level within the CNS group (Braem et al., 2011). The results obtained thus indicated that *S. xylosus* was well-adapted to the specificities of the recipe and technology applied in the experiment. This may partially be ascribed to the very moderate pH levels, taking into consideration that *S. xylosus* habitually prevails at such conditions (Cocolin et al., 2001a,b; Blaiotta et al., 2004; Iacumin et al., 2006) and possesses great ability in adapting to the meat environment (Mauriello et al., 2004; Drosinos et al., 2005; Planchon et al., 2006; Vermassen et al., 2014, 2016a,b; Leroy et al., 2017). This staphylococcal species is commonly isolated from different ecosystems and is known to adapt to various substrates (Nagase et al., 2002; Shale et al., 2005; Dordet-Frisoni et al., 2007).

As a second pattern, it became clear that added cultures of *S. saprophyticus* FPS1 and *S. equorum* DFL-S19 were only present in high proportions at the day of inoculation and were subsequently outcompeted by naturally occurring *S. xylosus* strain(s), once more suggesting their reduced fitness above the transition point of pH 5.3. The ability of the starter cultures to compete and overrule the background microbiota is a key criterion for starter culture selection and is not always guaranteed (Fonseca et al., 2013c; Stavropoulou et al., 2018a). For several CNS strains that are selected as starter cultures based on desirable metabolic traits, failure to survive and prevail in real food matrices has been reported (Villani et al., 2007; Sánchez Mainar and Leroy, 2015). Even the highly adapted and recurrent species of *S. equorum* and *S. saprophyticus* can thus become disadvantaged under certain production scenarios.

Coagulase-negative staphylococci are a metabolically heterogeneous group and can thus affect the quality of the end-products (Sánchez Mainar et al., 2017). During the present study, the volatile analysis indicated differences between the control batch and the inoculated ones as well as between the batches that were inoculated with different CNS strains, especially for the metabolites arising from amino acid conversion. Branched-chain amino acid-derived metabolites produced by CNS, such as 3-methyl butanol and 3-methyl butanoic acid, are commonly found in dry fermented sausages and play an important role in the typical aroma of those products (Beck et al., 2004; Ravyts et al., 2010). However, their production *in vitro* or/and *in situ* differs among species and even strains (Ravyts et al., 2010; Stavropoulou et al., 2015). The addition of the starter culture strains modified the volatile profiles, even when the inoculated strains were not the prevailing ones at the end of the ripening process. In previous studies, it has been demonstrated that non-dominant CNS species can sometimes be more metabolically active than the dominant ones, thus contributing to the quality traits of the end-product (Greppi et al., 2015).

In conclusion, the species diversity of CNS in fermented meats is variable and depends on the processing conditions applied. Among the different influencing factors, the raw materials harboring the background microbiota and the degree of acidification are of particularly relevance. The present study demonstrated through a combination of experimental set-ups that the pH could finetune the relative occurrence of the three main CNS species spontaneously associated with sausage fermentation, making abstraction of *S. carnosus* that mostly seems relevant as a starter culture in highly acidified fermented meats. Of course, the findings related to the effect of pH could not be completely untangled from the other conditions that prevail in the meat matrix, with a particular need to further explore the effects of curing salt, temperature, oxygen gradients, spices, and water activity. In addition, and despite the fact that the findings seemed rather robust on species level, intraspecies variability merits further investigation.

## Author Contributions

DAS contributed to the experimental work, the acquisition, processing, and interpretation of the data, and the drafting of the manuscript. HDM, AB, BJ, and PF contributed to the experimental work and the acquisition, processing, and interpretation of the data. LDV and SDS contributed to interpretation of the data and the drafting of the manuscript. FL contributed to the design of the work plan, the interpretation of the data, and the drafting of the manuscript.

## Conflict of Interest Statement

The authors declare that the research was conducted in the absence of any commercial or financial relationships that could be construed as a potential conflict of interest.

## Footnotes

1 https://www.nist.gov
