# Supplementary material for: Species Pervasiveness Within the Group of Coagulase-Negative Staphylococci Associated With Meat Fermentation Is Modulated by pH
Source: Front Microbiol. 2018 Sep 19;9:2232. doi: 10.3389/fmicb.2018.02232 (PMC6156374; doi:10.3389/fmicb.2018.02232)
Supplement: Supplementary file 1 [file Table_1.DOCX]

Supplementary Material

Species pervasiveness within the group of coagulase-negative staphylococci associated with meat fermentation is modulated by pH

Despoina Angeliki Stavropoulou ^1^, Hannelore De Maere ^2^, Alberto Berardo^3^, Bente Janssens^1^, Panagiota Filippou^1^, Luc De Vuyst^1^, Stefaan De Smet^3^, Frédéric Leroy^1*^

*** Correspondence:** Prof. Dr. ir. Frédéric Leroy: [Frederic.Leroy@vub.be](mailto:Frederic.Leroy@vub.be)

## Supplementary Table

**Supplementary Table 1.** Overview of the volatile compounds detected throughout the production process, expressed as arbitrary units (the area of each compound x 10^5^). In the first column, values in parenthesis indicate the presence of the different compounds in the meat batter after inoculation. Batches with (A) no coagulase-negative staphylococci added or inoculated with (B) *Staphylococcus xylosus* 2S7-2, (C) *Staphylococcus equorum* DFL-S19, or (D) *Staphylococcus saprophyticus* FPS1.

**Supplementary Table 1**

|  |  | **Time (days)** | | | | | |
| --- | --- | --- | --- | --- | --- | --- | --- |
| **Compound** | **Batch** | **1** | **2** | **7** | **14** | **21** | **28** |
| **Acetic acid**  (55.00 ± 32.00) | A  B  C  D | 50.02 ± 5.56  74.20 ± 11.40  39.40 ± 8.99  28.00 ± 7.58 | 88.0 ± 67.0  182.00 ± 101.00  147.00 ± 99.40  102.00 ± 95.10 | 21.10 ± 7.17  37.40 ± 5.50  47.90 ± 10.70  35.50 ± 11.60 | 104.00 ± 18.30  223.00 ± 40.90  230.00 ± 55.70  200.00 ± 51.90 | 160.00 ± 120.00  342.00 ± 97.90  372.00 ± 66.10  381.00 ± 219.00 | 75.40 ± 40.50  134.00 ± 31.40  198.00 ± 60.50 |
| **Acetoin**  (2.40 ± 1.60) | A  B  C  D | 7.34 ± 1.28  12.30 ± 13.20  n.d.  12.60 ± 7.25 | 9.90 ± 8.10  18.20 ± 14.50  20.70 ± 7.94  115.00 ± 14.40 | 18.60 ± 4.21  8.81 ± 0.72  4.69 ± 0.95  57.70 ± 14.20 | 26.50 ± 1.32  407.00 ± 63.70  377.00 ± 75.60  282.00 ± 28.70 | 4.94 ± 0.68  9.81 ± 5.05  4.38 ± 2.15  62.20 ± 35.50 | 30.20 ± 12.10  109.00 ± 35.60  55.70 ± 3.03 |
| **Benzaldehyde**  (10.00 ± 2.30) | A  B  C  D | 8.13 ± 0.12  10.70 ± 1.84  11.70 ± 7.70  13.90 ± 1.11 | 18.0 ± 11.0  25.60 ± 29.90  17.30 ± 5.44  19.30 ± 6.19 | 13.10 ± 0.37  13.10 ± 3.41  8.07 ± 1.77  13.60 ± 0.58 | 13.70 ± 0.10  10.20 ± 1.49  6.31 ± 0.12  8.53 ± 1.89 | 24.40 ± 3.08  42.70 ± 38.60  17.40 ± 2.41  39.00 ± 1.44 | 29.00 ± 19.80  20.90 ± 5.67  6.06 ± 1.17  n.d. |
| **Butanedione, 2,3-**  (10.00 ± 0.15) | A  B  C  D | 8.59 ± 0.45  9.21 ± 7.12  3.69 ± 0.56  14.30 ± 8.32 | 4.20 ± 3.40  5.49 ± 3.82  7.53 ± 1.75  31.20 ± 3.83 | n.d.  n.d.  n.d.  n.d. | 14.90 ± 3.45  175.00 ± 17.80  194.00 ± 24.70  152.00 ± 8.58 | 2.57 ± 1.31  1.64 ± 0.73  0.56 ± 0.14  14.4 ± 11.00 | 8.03 ± 3.57  16.10 ± 8.06  10.1 ± 1.77  20.9 ± 11.5 |
| **Butanone, 2-**  (2.20 ± 1.10) | A  B  C  D | 0.57 ± 0.53  0.81 ± 0.84  0.48 ± 0.34  0.51 ± 0.09 | 2.1 ± 1.1  0.39 ± 0.20  1.83 ± 0.58  0.98 ± 1.02 | n.d.  n.d.  n.d.  n.d. | 2.82 ± 1.30  4.72 ± 3.45  0.84 ± 0.61  0.86 ± 1.15 | 0.51 ± 0.47  1.33 ± 1.04  0.85 ± 0.60  7.25 ± 3.35 | 0.95 ± 1.03  0.32 ± 0.22  0.53 ± 0.59  1.50 ± 0.42 |
| **Butanoic acid**  (19.00 ± 1.90) | A  B  C  D | 48.00 ± 10.60  32.00 ± 6.99  36.40 ± 3.86  33.80 ± 2.45 | 24.0 ± 5.5  14.90 ± 1.45  20.4 ± 3.35  18.90 ± 6.32 | 15.30 ± 2.58  15.90 ± 1.42  20.60 ± 1.42  16.00 ± 1.20 | 34.90 ± 7.31  36.00 ± 0.21  32.40 ± 7.28  34.80 ± 10.20 | 11.20 ± 7.20  13.80 ± 4.09  10.00 ± 2.10  30.50 ± 7.50 | 12.10 ± 0.36  11.20 ± 1.72  31.20 ± 2.28  13.40 ± 1.97 |

**Supplementary Table 1** (*Continue*)

|  |  | **Time (days)** | | | | | |
| --- | --- | --- | --- | --- | --- | --- | --- |
| **Compound** | **Batch** | **1** | **2** | **7** | **14** | **21** | **28** |
| **Butanoic acid, 2-methyl**  (2.76 ± 0.45) | A  B  C  D | 7.85 ± 0.56  2.85 ± 0.25  4.02 ± 1.40  3.14 ± 0.70 | 1.76 ± 0.41  2.92 ± 0.85  2.76 ± 1.04  15.70 ± 3.46 | 3.39 ± 0.82  6.17 ± 1.31  5.05 ± 0.23  41.70 ± 5.37 | 20.40 ± 1.49  135.00 ± 14.90  156.00 ± 22.40  134.00 ± 22.50 | 3.87 ± 0.73  14.10 ± 6.29  2.96 ± 3.00  47.60 ± 37.00 | 7.13 ± 1.83  28.30 ±7.26  38.20 ± 4.22  44.60 ± 1.30 |
| **Butanoic acid, 3-methyl**  (7.51 ± 1.86) | A  B  C  D | 7.24 ± 2.16  8.87 ± 1.02  4.03 ± 0.45  4.81 ± 0.54 | 25.50 ± 2.02  18.20 ± 28.30  2.33 ± 0.66  17.30 ± 3.32 | 5.41 ± 1.15  9.93 ± 0.82  9.18 ± 2.81  38.80 ± 6.09 | 24.40 ± 1.69  76.70 ± 7.83  169.00 ± 20.30  155.00 ± 22.30 | 4.87 ± 1.65  20.00 ± 11.20  27.20 ± 33.00  82.60 ± 13.30 | 14.80 ± 5.26  39.90 ± 8.95  52.70 ± 7.59  59.90 ± 4.12 |
| **Butanol, 3-methyl**  (2.91 ± 0.56) | A  B  C  D | 11.90 ± 6.34  23.10 ± 5.47  32.70 ± 10.06  15.00 ± 4.62 | 14.10 ± 8.72  26.30 ± 13.20  10.10 ± 10.20  8.04 ± 4.20 | 3.48 ± 0.62  24.50 ± 3.37  5.44 ± 2.83  3.01 ± 0.92 | 33.40 ± 14.50  93.10 ± 9.42  88.60 ± 54.90  15.80 ± 0.95 | 60.00 ± 29.50  26.10 ± 10.40  34.10 ± 24.60  6.98 ± 0.80 | 16.60 ± 14.30  7.50 ± 2.91  n.d.  11.70 ± 8.89 |
| **Ethanol**  (n.d.) | A  B  C  D | 0.85 ± 0.37  1.81 ± 1.18  4.50 ± 3.66  2.71 ± 2.69 | n.d.  n.d.  n.d.  n.d. | n.d.  n.d.  n.d.  n.d. | 4.90 ± 2.97  2.96 ± 1.92  6.73 ± 3.05  4.73 ± 4.73 | n.d.  n.d.  1.81 ± 0.01  n.d. | n.d.  n.d.  n.d.  n.d. |
| **Ethyl acetate**  (n.d.) | A  B  C  D | n.d.  n.d.  n.d.  n.d. | n.d.  n.d.  n.d.  n.d. | n.d.  n.d.  n.d.  n.d. | 0.76 ± 0.35  n.d.  1.04 ± 0.65  2.20 ± 1.12 | n.d.  n.d.  n.d.  n.d. | 0.42 ± 0.25  1.32 ± 0.58  0.62 ± 0.52  0.28 ± 0.05 |
| **Heptadienal, 2,4-**  (n.d.) | A  B  C  D | 3.92 ± 3.27  13.3 ± 4.13  20.40 ± 3.85  2.90 ± 1.44 | 5.0 ± 2.7  11.10 ± 4.21  11.60 ± 4.48  5.52 ± 1.47 | n.d.  3.40 ± 1.10  2.08 ± 1.31  3.67 ± 0.17 | 5.57 ± 3.58  2.45 ± 3.33  3.30 ± 4.34  2.22 ± 3.45 | 47.60 ± 20.90  18.00 ± 2.61  37.80 ± 7.94  0.93 ± 1.24 | 7.71 ± 4.10  20.20 ± 6.46  n.d.  12.80 ± 4.73 |
| **Heptanol, 1-**  (1.60 ± 0.40) | A  B  C  D | 3.70 ± 1.90  6.35 ± 4.10  9.80 ± 7.84  1.63 ± 1.07 | n.d.  n.d.  n.d.  n.d. | n.d.  3.71 ± 0.88  1.89 ± 2.06  n.d. | 5.53 ± 2.38  2.14 ± 0.97  2.89 ± 1.30  1.10 ± 0.85 | 1.86 ± 1.14  8.58 ± 10.00  4.74 ± 3.80  1.90 + 1.18 | 2.41 ± 1.54  7.83 ± 5.08  1.28 ± 0.41  2.98 ± 1.97 |

**Supplementary Table 1** (*Continue*)

|  |  | **Time (days)** | | | | | | | | | | |
| --- | --- | --- | --- | --- | --- | --- | --- | --- | --- | --- | --- | --- |
| **Compound** | **Batch** | **1** | **2** | | **7** | | **14** | | | **21** | **28** | |
| **Hexanal**  (35.00 ± 13.00) | A  B  C  D | 130.00 ± 89.80  275.00 ± 82.00  354.00 ± 90.40  130.00 ± 25.40 | 290.00 ± 96.00  462.00 ± 218.00  297.00 ± 17.40  213.00 ± 58.00 | | 33.70 ± 12.40  108.00 ± 51.80  103.00 ± 55.50  130.00 ± 35.00 | | 483.00 ±262.00  66.90 ± 44.50  45.90 ± 60.90  51.00 ± 60.00 | | | 770.00 ± 330.00  370.00 ± 152.00  554.00 ± 352.00  99.30 ± 74.00 | 455.00 ± 169.00  515.00 ± 243.00  23.00 ± 22.90  417.00 ± 48.30 | |
| **Hexanoic acid**  (17.00 ± 2.00) | A  B  C  D | 47.50 ± 1.38  83.80 ± 40.90  92.30 ± 52.50  43.90 ± 9.88 | 26.0 ± 6.6  57.20 ± 36.50  40.00 ± 7.60  25.30 ± 8.73 | | 13.30 ± 0.92  14.50 ± 0.73  15.90 ± 0.88  17.80 ± 2.01 | | 93.00 ± 24.80  78.90 ± 19.80  114.00 ± 27.60  56.70 ± 3.17 | | | 397.00 ± 161.00  169.00 ± 7.70  531.00 ± 177.00  37.40 ± 18.20 | 51.70 ± 16.60  168.00 ± 105.00  42.50 ± 0.52  60.60 ±15.10 | |
| **Hexanoic acid, ethyl ester**  (0.26 ± 0.12) | A  B  C  D | 0.21 ± 0.00  0.48 ± 0.25  1.27 ± 0.79  1.09 ± 0.39 | n.d.  n.d.  n.d.  n.d. | | n.d.  n.d.  n.d.  n.d. | | 1.17 ± 0.11  0.94 ± 0.14  2.81 ± 1.04  1.14 ± 0.62 | | | 0.83 ± 0.09  0.31 ± 0.04  1.19 ± 1.05  0.23 ± 0.10 | 0.75 ± 0.32  4.38 ± 2.43  0.10 ± 0.01  0.66 ± 0.20 | |
| **Hexanol, 1-**  (n.d.) | A  B  C  D | 3.16 ±1.21  6.56 ± 1.64  7.18 ± 2.08  3.96 ± 0.55 | 1.10 ± 1.06  3.53 ± 1.41  5.26 ± 7.43  1.94 ± 1.15 | | 5.00 ± 1.99  2.85 ± 0.57  1.70 ± 1.17  1.47 ± 0.63 | | 1.91 ± 3.36  18.20 ± 6.42  24.40 ± 9.72  8.47 ± 3.59 | | | 8.76 ± 6.21  3.44 ± 0.98  7.90 ± 3.70  1.41 ± 1.48 | 5.93 ± 1.45  3.36 ± 1.03  0.99 ± 0.53  2.28 ± 0.60 | |
| **Hexenal, 2-**  (n.d.) | A  B  C  D | 8.45 ± 6.55  19.00 ± 5.26  25.10 ± 2.93  7.53 ± 2.95 | 14.0 ± 1.8  18.10 ± 5.31  20.60 ± 3.86  10.60 ± 3.11 | | n.d.  10.30 ± 1.24  7.90 ± 3.47  6.53 ± 3.42 | | 16.10 ± 0.07  29.30 ± 22.30  n.d.  n.d. | | | 45.10 ± 16.90  17.60 ± 5.83  27.40 ± 11.80  4.02 ± 5.01 | 12.20 ± 5.23  11.60 ± 7.94  0.92 ± 0.04  12.70 ± 2.56 | |
| **Nonanal**  (9.10 ± 2.09) | A  B  C  D | 21.90 ± 5.68  68.70 ± 23.00  89.60 ± 64.10  26.50 ± 5.45 | 43.0 ± 28.0  93.40 ± 55.70  68.20 ± 23.80  41.60 ± 16.50 | | 19.20 ± 4.74  36.50 ± 5.37  21.20 ± 8.86  28.60 ± 8.38 | | 66.50 ± 27.10  20.20 ± 3.97  28.20 ± 12.80  14.60 ± 7.14 | | | 183.00 ± 49.20  172.00 ± 100.00  183.00 ± 73.20  13.10 ± 7.57 | 61.80 ± 29.60  112.00 ± 52.80  4.17 ± 1.94  65.50 ± 24.20 | |
| **Nonanoic acid**  (5.60 ± 3.20) | A  B  C  D | 23.90 ± 3.58  20.70 ± 4.05  33.70 ± 1.80  13.30 ± 3.99 | | 2.6 ± 1.2  4.39 ± 1.39  6.20 ± 1.74  2.21 ± 1.10 | | 1.06 ± 0.48  0.99 ± 0.28  0.86 ± 0.11  1.01 ± 0.20 | | 23.30 ± 1.29  19.10 ± 6.17  19.20 ± 4.90  19.10 ± 4.63 | 3.68 ± 0.91  7.48 ± 2.47  5.94 ± 2.27  2.74 ± 1.40 | | | 8.07 ± 1.08  10.60 ± 1.13  7.30 ± 1.03  4.28 ± 0.42 |

**Supplementary Table 1** (*Continue*)

|  |  | **Time (days)** | | | | | |
| --- | --- | --- | --- | --- | --- | --- | --- |
| **Compound** | **Batch** | **1** | **2** | **7** | **14** | **21** | **28** |
| **Octanal**  (2.90 ± 1.10) | A  B  C  D | 8.98 ± 5.61  35.40 ± 14.90  52.90 ± 35.80  9.88 ± 3.41 | 26.0 ± 17.0  58.30 ± 33.80  37.00 ± 11.20  25.80 ± 8.30 | 4.43 ± 1.21  11.20 ± 5.44  11.60 ± 5.94  9.72 ± 4.91 | 44.30 ± 24.10  7.21 ± 2.90  8.82 ± 8.86  6.13 ± 5.91 | 81.30 ± 20.30  73.60 ± 34.00  70.80 ± 37.70  5.74 ± 3.75 | 44.60 ± 18.90  57.80 ± 21.60  2.28 ± 1.22  41.70 ± 15.60 |
| **Octanoic** **acid**  (2.60 ± 1.20) | A  B  C  D | 11.40 ± 1.73  13.40 ± 1.53  15.40 ± 2.86  8.29 ± 1.69 | 3.5 ± 1.6  5.05 ± 1.63  5.55 ± 1.18  3.40 ± 1.19 | 2.68 ± 0.71  3.41 ± 0.39  3.45 ± 0.38  4.06 ± 0.46 | 22.90 ± 2.41  25.00 ± 0.57  28.30 ± 1.94  26.30 ± 0.92 | 16.70 ± 5.63  23.00 ± 10.50  25.90 ± 12.30  10.10 ± 3.23 | 11.90 ± 1.39  19.90 ± 4.55  14.30 ± 1.87  14.50 ± 0.88 |
| **Octanoic** **acid**, **ethyl** **ester**  (n.d.) | A  B  C  D | n.d.  n.d.  0.13 ± 0.07  n.d. | n.d.  n.d.  n.d.  n.d. | 0.33 ± 0.25  n.d.  n.d.  n.d. | 0.21 ± 0.00  0.33 ± 0.05  0.90 ± 0.25  0.54 ± 0.15 | n.d.  n.d.  0.33 ± 0.13  n.d. | n.d.  n.d.  n.d.  n.d. |
| **Octanol, 1-**  (n.d.) | A  B  C  D | 3.76 ± 0.90  7.78 ± 2.60  11.50 ± 10.00  4.01 ± 0.17 | 1.4 ± 0.94  2.96 ± 2.33  1.27 ± 0.54  1.12 ± 0.66 | 1.62 ± 0.73  3.07 ± 0.88  1.75 ± 0.28  1.84 ± 1.25 | 5.93 ± 0.60  2.82 ± 0.57  2.43 ± 1.68  1.93 ± 0.74 | 14.60 ±10.10  14.80 ± 20.80  7.54 ± 1.97  n.d. | 3.24 ± 1.46  5.99 ± 2.94  0.22 ± 0.01  3.72 ± 1.54 |
| **Octen-3-ol, 1-**  (n.d.) | A  B  C  D | n.d.  n.d.  n.d.  n.d. | 13.00 ± 8.4  33.60 ± 15.80  27.20 ± 5.16  16.10 ± 5.78 | 2.54 ± 1.42  37.70 ± 3.26  27.70 ± 12.80  29.80 ± 11.70 | n.d.  n.d.  n.d.  n.d. | 17.50 ± 18.30  88.20 ± 61.90  9.03 ± 0.58  n.d. | 7.39 ± 1.21  n.d.  n.d.  n.d. |
| **Pentanal**  (3.90 ± 2.00) | A  B  C  D | 13.10 ± 10.30  28.90 ± 6.76  41.70 ± 8.69  13.50 ± 0.97 | 38.0 ± 8.4  51.10 ± 29.50  38.20 ± 2.65  20.7 ± 8.56 | n.d.  1.94 ± 2.01  n.d.  1.22 ± 0.57 | 42.50 ± 26.60  10.60 ± 2.98  4.13 ± 4.32  7.46 ± 7.96 | 87.40 ± 44.90  39.10 ± 15.40  52.40 ± 36.10  6.07 ± 5.29 | 43.20 ± 14.50  47.20 ± 27.30  3.45 ± 3.46  41.20 ± 5.24 |
| **Pentanedione**, **2,3-** (8.90 ± 1.50) | A  B  C  D | 1.22 ± 0.21  0.86 ± 0.09  2.56 ± 1.31  n.d. | n.d.  n.d.  n.d.  n.d. | n.d.  n.d.  n.d.  n.d. | 1.56 ± 1.35  7.50 ± 0.84  7.30 ± 1.18  2.65 ± 1.17 | 1.57 ± 0.83  7.51 ± 7.21  0.66 ± 0.33  2.96 ± 1.11 | 4.66 ± 0.58  4.89 ± 1.40  2.78 ± 1.48  7.98 ± 0.43 |

**Supplementary Table 1** (*Continue*)

|  |  | **Time (days)** | | | | | |
| --- | --- | --- | --- | --- | --- | --- | --- |
| **Compound** | **Batch** | **1** | **2** | **7** | **14** | **21** | **28** |
| **Pentanol, 1-**  (2.80 ± 0.56) | A  B  C  D | 11.90 ± 6.29  21.90 ± 5.59  34.60 ± 10.20  12.00 ± 5.43 | 8.1 ± 8.8  18.10 ± 9.39  9.80 ± 10.60  4.47 ± 3.80 | 2.36 ± 0.72  6.65 ± 1.91  4.53 ± 1.59  4.51 ± 2.55 | 31.90 ± 14.10  63.10 ± 44.40  85.40 ± 53.20  15.30 ± 0.89 | 58.50 ± 28.40  25.00 ± 9.45  34.10 ± 24.60  3.29 ± 2.68 | 11.50 ± 13.90  7.43 ± 3.04  n.d.  4.02 ± 3.46 |
| **Penten-3-ol, 1-**  (0.34 ± 0.24) | A  B  C  D | 7.11 ± 0.70  8.75 ± 2.79  14.80 ± 0.90  5.12 ± 1.12 | 6.9 ± 5.4  6.29 ± 5.42  6.93 ± 5.42  6.04 ± 5.00 | 0.25 ± 0.06  5.34 ± 2.24  3.94 ± 0.89  3.71 ± 1.60 | 6.98 ± 2.79  10.50 ± 6.00  5.40 ± 2.86  3.82 ± 3.23 | 7.34 ± 7.97  6.99 ± 5.86  2.84 ± 3.97  0.79 ± 0.61 | 6.73 ± 1.21  7.25 ± 1.72  0.96 ± 0.09  5.62 ± 1.89 |
| **Phenylethyl alcohol** (n.d.) | A  B  C  D | 0.63 ± 0.09  0.75 ± 0.44  0.35 ± 0.06  n.d. | 0.60 ± 0.37  0.76 ± 0.33  0.13 ± 0.05  0.91 ± 1.29 | 1.84 ± 0.20  1.35 ± 0.09  0.85 ± 0.21  0.42 ± 0.07 | 10.90 ± 3.85  18.10 ± 5.32  21.00 ± 8.36  3.14 ± 0.82 | 1.96 ± 1.41  3.60 ± 0.67  2.31 ± 0.01  0.36 ± 0.37 | 1.77 ± 0.32  6.00 ± 0.81  2.23 ± 1.05  0.88 ± 0.04 |
| **Propanal, 3-(methylthio)-**  (n.d.) | A  B  C  D | n.d.  n.d.  n.d.  n.d. | 4.65 ± 0.33  3.92 ± 1.03  4.58 ± 0.40  4.27 ± 1.35 | 12.10 ± 0.16  6.88 ± 1.83  5.19 ± 2.03  5.59 ± 0.78 | 7.30 ± 1.04  5.91 ± 0.48  n.d.  4.31 ± 1.33 | n.d.  n.d.  n.d.  n.d. | 14.90 ± 7.54  5.11 ± 2.37  11.20 ± 2.98  6.82 ± 2.25 |
| **Propanoic acid, 2-methyl** (n.d.) | A  B  C  D | n.d.  n.d.  n.d.  1.50 ± 0.70 | 0.38 ± 0.24  0.80 ± 0.60  0.38 ± 0.22  0.72 ± 0.54 | 0.39 ± 0.07  0.61 ± 0.12  0.54 ± 0.11  1.34 ± 0.36 | 2.85 ± 0.22  9.40 ± 2.09  9.66 ± 1.89  5.30 ± 0.82 | n.d.  0.28 ± 0.16  n.d.  2.19 ± 0.69 | 0.72 ± 0.23  1.16 ± 0.52  2.03 ± 0.35  1.40 ± 0.16 |
| **Propanol, 2-methyl** (n.d.) | A  B  C  D | n.d.  2.67 ± 1.72  3.09 ± 1.63  n.d. | n.d.  n.d.  n.d.  n.d. | n.d.  n.d.  n.d.  n.d. | n.d.  n.d.  n.d.  n.d. | 22.30 ± 6.57  11.90 ± 9.28  3.22 ± 2.48  n.d. | 3.60 ± 1.68  n.d.  n.d.  6.67 ± 2.08 |
| **Undecenal, 2-**  (n.d.) | A  B  C  D | n.d.  1.79 ± 1.11  3.64 ± 4.35  n.d. | n.d.  4.62 ± 5.03  2.52 ± 0.85  1.21 ± 1.53 | n.d.  0.56 ± 0.23  0.25 ± 0.15  n.d. | 2.00 ± 0.19  n.d.  n.d.  n.d. | 11.10 ± 2.93  30.90 ± 39.20  15.60 ± 5.94  n.d. | 3.25 ± 0.63  8.48 ± 5.21  n.d.  n.d. |

n.d.: not detected
